# Crush syndrome diagnosis and management in resource-constrained settings: A Delphi study

**DOI:** 10.1371/journal.pone.0331596

**Published:** 2025-09-02

**Authors:** Smitha S. Bhaumik, Julia Finn, Chelsie Fleischer, Elmin Steyn, Hendrick J. Lategan, Julia M. Dixon, Anna M. Maw, Nee-Kofi Mould-Millman

**Affiliations:** 1 Department of Emergency Medicine, University of Colorado Denver, Anschutz Medical Campus, Aurora, Colorado, United States of America; 2 Department of Surgical Sciences, Division of Surgery, Stellenbosch University, Cape Town, Western Cape Province, South Africa; 3 Department of Medicine, University of Colorado Denver, Anschutz Medical Campus, Aurora, Colorado, United States of America; University of Zagreb School of Medicine: Sveuciliste u Zagrebu Medicinski fakultet, CROATIA

## Abstract

**Background:**

Crush syndrome is an important source of morbidity and mortality in resource-constrained settings including earthquake disaster zones, austere military environments, and countries where motor vehicle collisions and interpersonal violence are prevalent. In South Africa and other countries with high rates of community violence, patients develop crush due to a unique form of trauma called community assault, where individuals suspected of wrongdoing are assaulted by multiple persons as a form of mob justice. The purpose of this study is to generate consensus about crush syndrome definitions and endpoints to inform the development of scoring systems appropriate for community assault and usable in resource-limited settings.

**Methods:**

This study used in-depth interviews of clinicans from South Africa to determine the challenges associated with crush management in resource-constrained environments. These qualitative findings informed a subsequent Delphi survey process which sought consensus regarding crush definitions, endpoints, and co-variates. Three surveys were administered to an international panel of clinicians with experience managing crush injuries in military, disaster, and civilian clinical environments. There was a pre-established consensus threshold of 75%.

**Results:**

There were 8 interview participants and 15 Delphi participants. These clinicians recommended maintaining a high index of suspicion for crush syndrome as this diagnosis can easily be overlooked in polytrauma patients, and advised early administration of intravenous fluids titrated to urine output and respiratory status. Crush injury was conceptualized as a localized process of muscle injury from trauma, whereas crush syndrome was viewed as the resulting systemic complications including renal failure and hemodynamic instability. Preferred clinical endpoints included acute kidney injury, renal replacement therapy, and need for respiratory support.

**Conclusion:**

This study provided context related to crush injury management in resource-constrained environments. Clinical risk prediction models must account for the unique patient populations and data limitations commonly encountered in these settings.

## Introduction

Crush injury, also known as traumatic rhabdomyolysis, occurs when an individual experiences prolonged pressure or compression of a muscle group. [1] This is a common source of morbidity among earthquake and blast victims but can also be a sequela of interpersonal violence. [2–4] In crush injury, muscle cells lyse and release intracellular contents into the circulation. In severe cases, these proteins and enzymes cause systemic complications, including renal failure, hemodynamic instability, electrolyte disturbances, and cardiac dysrhythmias. [5] Patients who develop these systemic findings are diagnosed with crush syndrome and require aggressive resuscitation; this can include intravenous fluids, respiratory support, renal replacement therapy (RRT), and other critical care. [6]

Crush injury cases in South Africa are predominantly due to a unique form of trauma called community assault. [7] In community assault, multiple persons beat an individual with canes and whips, targeting the muscles of the back and lower extremities. [8] Victims may also sustain concomitant traumatic brain injury, visceral organ injury or skeletal fractures. [9] Community assault is a form of vigilante justice carried out by community members who want to punish a perpetrator of crime and many cases result in permanent disability and death. [8,10] This phenomenon has been observed in many regions, including East Africa, Central and South America, and South Asia. [11–15] Historically, clinical algorithms for crush syndrome have not included patients injured by community assault. [16,17]

The early identification of crush syndrome is challenging in low-resource environments where there are limited diagnostic tools. However, for patients with severe manifestations, timely transfer to hospitals with critical care and dialysis capabilities is imperative. Trauma experts assert that many cases of crush syndrome are missed on initial presentation, and that delays to diagnosis worsen morbidity and mortality. [18,19] Delayed diagnosis of crush syndrome also has potential economic implications, given the expense associated with renal care in overburned health systems. For example, a study across 31 countries found that the median yearly cost of treatment was USD $4568 for acute kidney injury and USD $49,926 for hemodialysis per patient. [20]

The purpose of this study is to generate expert consensus about crush syndrome diagnosis and management in resource-limited settings. This information is intended to inform the development of scoring systems for use at the point of care. Such tools may help with treatment and disposition decision making for patients with common mechanisms of crush injury, including community assault. [21]

## Methods

This study used qualitative interviews and Delphi surveys of clinical experts to achieve the following aims: (1) Describe the challenges associated with the clinical care of patients with crush injury in resource-constrained settings; (2) Define the terms crush injury and crush syndrome; (3) Identify diagnostic tests that are practical for use in resource-constrained settings and (4) Define the endpoints for crush syndrome that are of clinical importance. The key output is a proposed clinical algorithm that clinicians can reference when determining patient disposition, and the desired outcome is improved uniformity in crush syndrome diagnosis and management in low-and-middle country (LMIC) settings. [22] This algorithm will be further refined and validated in future studies.

For aim one, authors (SB, JD) performed in-depth interviews of South African clinicians who treat patients with crush injury within a tiered, resource-constrained public hospital system in the Western Cape Province. Both interviewers are US-trained academic emergency medicine physicians with prior qualitative interviewing and global health research experience. Participants were identified as a convenience sample of clinicians known to the investigators from prior research collaborations and contacted by email. A snowball purposive sampling technique was used to identify additional clinicians for interview from a broad range of specialties and clinical practice environments. The interview guide (see S1 Appendix) was pilot tested using physicians on the study team as mock participants. The private semi-structured interviews were conducted through video conferencing from May 1, 2023 through August 31, 2023. Interviews were audio recorded, transcribed, and coded by two independent members of the team (AM, CF) using Dedoose software (Dedoose v9.2.006, Los Angeles CA, 2023). Interviews concluded once thematic saturation was reached for the major categories noted on the interview guide. Two team members (SB, JF) read through all transcripts and codes independently and discussed in person to form consensus around major categories and themes using inductive thematic analysis. [23] Findings were collated into a pictorial display and synthesized in summary paragraphs.

Aims two through four were addressed through a Delphi consensus-building process. [21] Authors assembled an international panel of clinician experts who had experience treating crush injury in LMIC civilian health systems, and/or in disaster and austere military settings. A convenience sample was used, consisting of individuals known to the authors and others identified through a literature review, all of whom had relevant expertise in crush syndrome management. Candidates for the Delphi process were emailed and formally invited to participate; in total, 18 individuals were invited, and 15 chose to participate.

The Delphi participants completed three consecutive rounds of asynchronous online surveys from October 1, 2023 through June 31, 2024. [24,25] The first survey included open-ended questions developed using aim one themes. The second survey included summaries of the round one responses and participants voted to arrive at consensus, defined *a priori* as 75% of participants voting for the same option. In the third survey, participants were presented with the consensus statements that were generated from prior rounds, prompted for assent, and given the option for final comment. Ordinal data from the Delphi surveys were summarized as means. This study underwent ethics review by the Colorado Multiple Institutional Review Board and received a certificate of exemption (COMIRB # 22–1274). All participants received a written consent form outlining study risks and benefits; interview participants provided verbal informed consent to the interviewer, and Delphi participants provided written informed consent on the first survey form, as approved by the ethics board.

## Results

### Phase 1: Qualitative Interviews with South African clinicians treating crush injury

**Fig 1:**
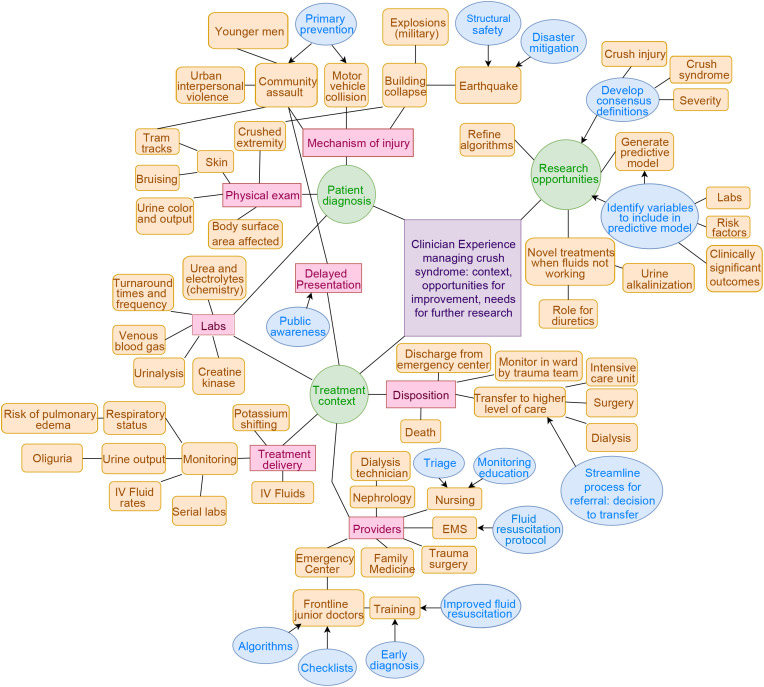
Themes from in-depth interviews regarding clinical context for crush injury in South Africa. Green represents major categories, pink represents sub-categories, and orange represents individual themes. Blue represents areas for quality improvement.

The qualitative interviews included 8 South African clinicians (3 women, 5 men), including specialists in emergency medical services (n = 1), family medicine (n = 2), emergency medicine (n = 3), trauma surgery (n = 1), and nephrology (n = 1). Major themes are depicted in **Fig 1**, and representative quotes are included throughout.

#### 1.1 Crush patient profile.

Participants spoke from personal experience caring for victims of crush injury in the Western Cape health system. Typical patients with crush injury were described as young, previously healthy males without significant comorbidities. Victims of community assault and pedestrians and motorists injured in motor vehicle collisions were noted as the most common groups with crush injury. Clinicians believed community assault patients generally came from disenfranchised areas with high rates of interpersonal violence. These patients were frequently abandoned in inaccessible areas, with prehospital transport delayed by several hours. Clinicians felt that patients with crush injury generally had a good prognosis when identified early, but noted this diagnosis is sometimes missed or delayed.

*“With community assault, they’re often more easily identified because of the nature of what happened to them…it [crush injury] is thought of quicker… the problem comes in where we’ve had patients with pedestrian vehicle accidents who go for pan scans, etcetera, but everyone has missed that this patient is a potential crush injury patient.” – Participant 2*

#### 1.2 Crush diagnostic approach.

**Fig 2:**
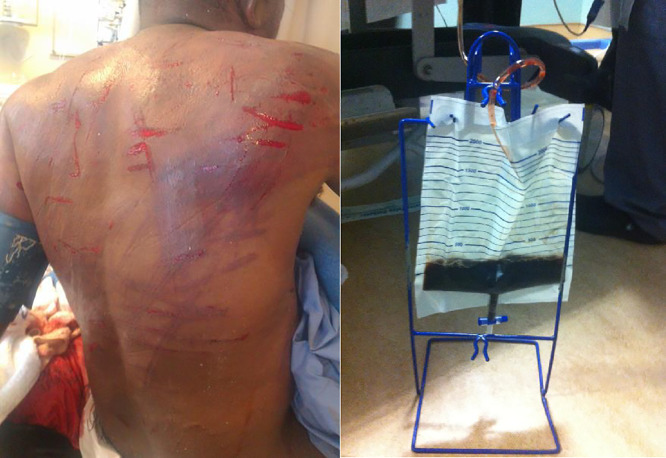
Physical exam findings in community assault. The image on the left shows “tram tracks” from sjambok whip injury. The image on the right shows “Coca Cola” colored urine in a patient with crush syndrome. Patient permission was obtained to share these de-identified images.

The diagnostic approach to crush injury varied based on available hospital resources. Family medicine physicians who staff rural hospitals rely on the physical exam and urinalysis to diagnose crush injury; they look for bruising, linear skin markings called ‘tram tracks’, and ‘Coca Cola’ discoloration of the urine (see **Fig 2**). Point-of-care venous blood gas tests and serum chemistries are also used to evaluate for crush syndrome with metabolic acidosis, hyperkalemia, and elevated serum creatinine being suggestive of this entity. Regional and district hospitals used these same diagnostic approaches, with addition of serum creatine kinase testing to further stratify crush severity.

#### 1.3 Crush treatment approach.

Fluid resuscitation with crystalloids was described as the cornerstone of crush injury management. Participants felt it was important to titrate intravenous fluid rates based on urine output, starting with higher rates (e.g., 250 mL/hour) in the early phase of resuscitation and then decreasing rates over time. Transurethral catheters (i.e. Foley’s catheters) were commonly inserted to assist with urine output monitoring. Clinicians cautioned that patients with delayed presentations were at greater risk for renal failure and may develop pulmonary edema with high rates of infusion. They advised that fluid infusion rates be adjusted based on the degree of oliguria and by using serial pulmonary exams. Participants noted that emergency department overcrowding and healthcare provider shortages made it difficult to monitor rates of fluid administration to the degree that is desired. Medications for potassium shifting were used for the subset of crush syndrome patients with hyperkalemia. Cited medications included furosemide, combined insulin/dextrose, and salbutamol.

*“For us it’s fluid, fluid, fluid…to get the myoglobin out of the kidneys… [If a patient] comes in and he is still to be triaged and we suspect it might be crush, we would just try to get a drip up as soon as possible to get him some fluids.” – Participant 8*

In terms of admission and transfer decisions, participants stated that within the South African tiered health system where they practice, patients with isolated crush injury are often discharged from the emergency department, whereas those with moderate-to-severe crush syndrome are referred to trauma surgery for hospitalization. Lastly, patients who develop renal failure are transferred to the tertiary hospital for nephrology consultation and possible dialysis.

#### 1.4 Opportunities for quality improvement.

Participants recommended maintaining a high index of suspicion for crush syndrome among patients with polytrauma and revisiting this diagnosis following acute resuscitation for more time-sensitive injuries. Suggested quality improvement activities included training modules on crush diagnosis and fluid resuscitation for junior doctors, checklist algorithms to standardize treatment, nursing education on triage and monitoring of crush patients, and development of a prediction tool for early identification of crush patients who may require intensive care. Staff and equipment shortages were also a common concern.

*“I mean sometimes we do not even have IV pumps to monitor the rate of the infusion for the fluids that are going in or the urine output isn’t monitored… nursing staff being understaffed, the doctors being understaffed, just the patient load. So just those little things, even though we would want to say we want to reevaluate the patient hourly, it would not be a practical step in how things are at the moment.” – Participant 5*

### Phase 2: Delphi study with international panel of clinicians treating crush injury

#### 2.1 Delphi participant demographics.

There were 15 participants in the Delphi study, including 10 men and 5 women. All 15 participated in the first survey and 14 participated in rounds two and three. Specialties represented include emergency medical services, emergency medicine, family medicine, trauma surgery, nephrology, and forensic pathology. Four participants had prior military experience, and four had prior experience with disaster response (e.g., Haiti earthquake, Turkiye-Syria earthquake). Nationalities represented included South Africa (n = 10), USA (n = 2), Turkiye (n = 2) and Congo (n = 1). All participants had treated at least 10 cases of crush injury, and over half estimated having treated greater than 50 cases.

#### 2.2 Consensus findings from the Delphi process.

##### 1. Definition of crush injury

Participants were presented with multiple definitions of crush injury and given the option of providing their own personal definition. Ultimately, consensus was reached for the following definition provided by Haines and Doucet [26](86% consensus):

*Crush injury is the local manifestation of direct physical trauma, and can present as muscle injury and swelling, along with possible muscle necrosis and neurologic dysfunction in the affected areas. It can be due to the primary direct effect of trauma or ischemia-reperfusion injury related to compression.*

##### 2. Definition of crush syndrome

Participants modified pre-existing definitions to define crush syndrome as follows (78% consensus):

*Crush syndrome is the systemic manifestation of extensive skeletal muscle damage due to the disruption of cellular integrity and release of its contents into circulation. It manifests as hemodynamic and metabolic disturbances, and can result in acute kidney injury, multisystem organ dysfunction, or death.*

##### 3. Crush syndrome as a spectrum of disease

Participants viewed crush syndrome as a spectrum of disease (93% consensus) and categorized the syndrome as mild, moderate and severe. Classification schemes were based on laboratory parameters (e.g., creatine kinase levels, severity of renal impairment), physical exam findings (e.g., pulmonary edema, oliguria), and/or intensity of resource utilization (e.g., intravenous fluids, renal replacement therapy, mechanical ventilation, surgical treatments such as fasciotomy, debridement or amputation). Broadly, experts classified mild crush syndrome as being self-limited, typically requiring intravenous fluids alone, whereas moderate-to-severe crush syndrome required longer periods of hospitalization, more aggressive intervention, and carried higher mortality risk.

##### 4. Diagnostic and prognostic utility of laboratory tests for crush syndrome

**Fig 3:**
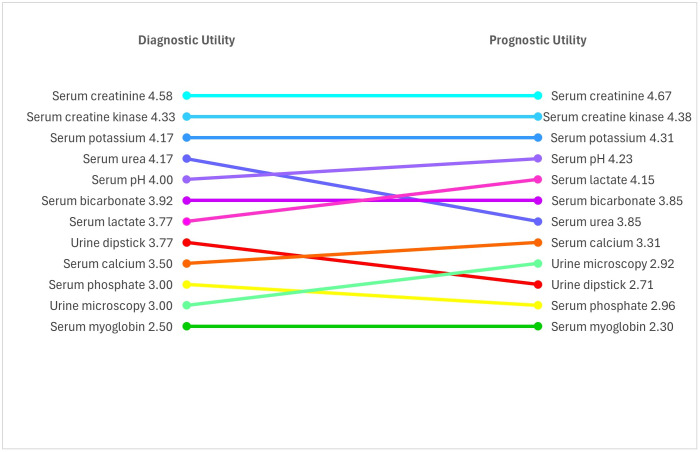
Diagnostic and prognostic utility of laboratory tests for crush syndrome.

Participants indicated the usefulness of each laboratory test on a scale from 1 (not useful) to 5 (extremely useful) (**Fig 3**). They created separate rankings based on diagnostic and prognostic utility for crush syndrome. Of note, participants indicated that non-laboratory measures such as urine output (mean score 4.57) and EKG (mean score of 3.43) also carried prognostic value.

Participants described use of serial urine dipsticks to assess for myoglobinuria, point-of-care venous blood gas measurements, and monitoring of urine output as practical ways to monitor patients with crush injury in resource-limited settings. If the patient has poor urine output, severe metabolic acidosis, or persistent gross hematuria despite fluid resuscitation, this could signal renal dysfunction.

##### 5. Clinical predictive tools: timing and outcomes

Participants recommended using clinical data from the first 4–6 hours of the emergency department visit (85% consensus) in future clinical predictive tools. The tool should facilitate emergency department disposition by predicting organ dysfunction in the following rank order: (1) acute kidney injury, (2) need for dialysis, (3) need for respiratory support (i.e., hypoxia requiring oxygen administration), and (4) need for aggressive fluid resuscitation (i.e., hypotension requiring intravenous fluids). Health systems outcomes such as need for hospital admission, intensive care unit admission, and in-hospital mortality, were less favored (ranked #5, #6 and #7, respectively). Most participants preferred use of a composite outcome (78% consensus).

“*A tool that can help facilities prognosticate would assist facilities to identify the need for transfer as soon as possible. For instance, any facility can provide respiratory or circulatory support, but specialized care like renal replacement therapy and intensive care unit admission needs advance knowledge and arrangement to improve outcomes.” – Delphi survey 1 anonymous comment*

#### 2.3 Additional noteworthy findings from the Delphi process.

##### 1. Disposition decisions for crush injury

The Delphi participants offered several suggestions for patient disposition. These have been collated and summarized in **Table 1**, and are also shared in a clinical algorithm format in Supplemental materials S2 Appendix.

**Table 1:** Delphi expert suggestions for clinical risk stratification of patients with crush injury in resource-constrained settings.

Crush Syndrome Severity	Mechanism of Injury	Physical Exam Findings	Initial Vitals	Urine Output	Labs/EKG	Other Indicators
Minor crush syndrome that can be safely discharged from Emergency Center	• Minor mechanism of injury	• Soft muscle compartments without severe bruising • Ambulatory	• Normal Glasgow Coma Scale (GCS)	• Voiding freely with clear urine	• Labs without significant derangements	• Minimal analgesic requirements
Crush syndrome warranting hospitalization	• Moderate to severe mechanism of injury • Extensive blunt trauma • Prolonged ischemia from tourniquet or vascular injury	• Muscle weakness • Large areas of muscle bruising or swelling • Pain out of proportion to physical exam	• Abnormal GCS • Tachycardia • Bradycardia • Hypotension • Signs of respiratory distress	• Poor urine output • Hematuria or ‘Coca Cola’ coloured urine	• Abnormal pH • Abnormal CK • Abnormal creatinine • Hyperkalemia	• Complicated anticipated course • Unable or unwilling to take oral fluids
Crush syndrome requiring dialysis	• Moderate to severe mechanism of injury • Extensive blunt trauma • Prolonged ischemia from tourniquet or vascular injury	• Generalized anasarca • Pulmonary edema • Altered mental status from uremia	• Respiratory distress requiring ventilatory support	• Anuric or with persistent oliguria	• Severe acidosis • Severe hyperkalemia • Symptomatic uremia (pericardial friction rub, frosting of the skin, encephalopathy) • Bradyarrhythmia in the setting of hyperkalemia	• None

##### 2. Body surface area assessment

Participants did not support the use of total body surface area calculations of bruised skin (e.g., akin to the rule of 9’s for burns) for predicting the severity of crush syndrome (7% fully supported, 29% supported with reservations, 64% not supported or not familiar with the tool). They warned that such measurements are subjective and lack standardization. Moreover, the severity of crush syndrome may not correlate with what is visible on the surface, as there may be deeper injury to the subcutaneous tissues. Rather, they suggested using the patient’s mechanism of injury and the occurrence of ischemia-reperfusion (e.g., tourniquet removal, history of limb entrapment) to raise one’s clinical suspicion that crush injury may be present.

##### 3. Time elapsed from injury to assessment

Time from injury to assessment was felt to be important both for laboratory interpretation and for patient outcome. Providers felt confident excluding crush syndrome based on normal laboratory investigations performed >6 hours post injury. In contrast, if a patient presents soon after injury, he/she is kept for observation and treated for presumed crush syndrome with intravenous fluids pending the results of serial laboratory investigations. Participants emphasized the importance of early intravenous fluid initiation and provided the following hypothetical example:

“*Time since injury is a critical factor. Two patients can arrive after community assault, both with CK [creatine kinase of] 7000, with crush injuries only. Patient A makes it to hospital within two hours post assault and is promptly fluid resuscitated. He does well and is discharged the next day. Patient B goes into hiding after the assault for more than 24 hours, without access to water, and finally makes it to hospital, markedly dehydrated. Despite aggressive fluid resuscitation he develops acute renal failure and requires renal replacement therapy.” – Delphi survey 1 anonymous comment*

## Discussion

This study examined crush syndrome diagnosis and management in resource-constrained settings. While crush syndrome is traditionally associated with mechanisms like earthquakes and explosions, there is an underappreciated burden within civilian high-trauma settings where community assault and motor vehicle collisions are prevalent [18,27].

Challenges specific to LMIC contexts noted by study participants include late patient presentations due to bystander delays in activating the emergency response system and limited availability of prehospital transport; emergency center staff and equipment shortages that make it difficult to closely monitor intravenous fluid administration rates and urine output; and limited access to dialysis, which is often only available at the tertiary center, necessitating interfacility transfer. Patients with crush injury from community assault may be particularly difficult to treat due to the presence of polytrauma. Fluid protocols for crush resuscitation may have to be altered, for example, in the presence of concomitant cerebral edema or pulmonary contusion.

The Delphi process identified consensus definitions for crush injury and crush syndrome and clinical endpoints for use in future tool development. As there are many overlapping definitions for crush in the literature, this study helps fill a void by creating a shared mental model [5,17,18]. Preferred clinical endpoints included acute kidney injury, need for dialysis, and need for respiratory support; these endpoints mirror those listed by the Eastern Association for the Surgery of Trauma working group on rhabdomyolysis. [28] The framework shared in Table 1 and S2 Appendix about indications for hospitalization and dialysis serves as a starting point for future planned research on clinical prediction tool development tailored for LMICs.

While there are many crush/rhabdomyolysis scoring tools, none have included patients with community assault, and most use laboratory measures that are not routinely collected in resource-constrained settings. The McMahon score for rhabdomyolysis is likely the best known tool used in clincial practice. Published in 2013, the McMahon score estimates the risk of renal replacement therapy and/or in-hospital mortality among patients admitted with rhabdomyolysis [16]. While the McMahon score has been externally validated, it may not be readily used in LMIC settings because it requires CK levels, which are not always available, and because it uses in-hospital mortality as a key outcome. The experts in our study did not recommend mortality as an endpoint in resource-constrained settings because mortality can be influenced by variations in care across practice settings, and because mortality in crush syndrome patients may be related to co-occurring traumatic injuries.

Several other predictive models have emerged alongside McMahon’s seminal work. [29–31]. These predictive models used admitted patients with rhabdomyolysis diagnosed based on CK value thresholds. These models use variables that are not routinely collected in resource-constrained settings (e.g., CK, SOFA score, AST, prothrombin time), which may limit their utility. Our study suggests that a scoring tool that incorporates plausible mechanisms of injury (including community assault), time elapsed from injury to presentation, and readily available laboratory values (e.g., serum creatinine, potassium, pH and bicarbonate), with a focus on objective endpoints (e.g., severe acute kidney injury, pulmonary edema with need for respiratory support) may be more suitable for South Africa and similar contexts.

This study has a few limitations. First, the qualitative interviews included a convenience sample of South African clinicians who practice in the Western Cape Province. This region sees large volumes of crush injury and these clinicians are very experienced managing this pathology. A second, related limitation is that 10 out of 15 participants in the Delphi study were from South Africa. The results of this Delphi study therefore may be most applicable to the South African context. Future research that includes clinican perspectives from other LMICs would help improve the generalizability of the findings. Additionally, the definition of community assault may require recalibration specific to each geographic area to account for regional differences in patterns of injury.

## Conclusions

In conclusion, we examined expert opinion on crush syndrome management in resource-constrained settings. Experts recommended that clinicians maintain a high index of suspicion for crush syndrome whenever there is a plausible injury mechanism or consistent physical exam findings. They advised early aggressive administration of intravenous fluids titrated to urine output and respiratory status, and recommended that time of injury be factored into the treatment plan, as patients with delayed presentations may be prone to fluid overload. Experts provided definitions, covariates and clinical endpoints that will be useful for predictive model development. Future research should focus on clinical risk stratification of crush syndrome patients using data points that are readily available in resource-constrained settings, as have been highlighted here. Such evidence-based risk stratification tools may improve patient outcomes through early identification of crush patients, timely initiation of fluid resuscitation, and recognition of the subset who will require emergent dialysis and interfacility transfer.

## Supporting information

S1 Appendix Interview guide for qualitative interviews conducted with South African clinicians experienced in crush injury management. (DOCX)

S2 Appendix Proposed clinical algorithm for patients with crush injury. (PDF)

## References

[1] Smith J, Greaves I. Crush injury and crush syndrome: a review. J Trauma. 2003;54(5 Suppl):S226-30. doi: 10.1097/01.TA.0000047203.00084.94 12768130

[2] Long B, Liang SY, Gottlieb M. Crush injury and syndrome: a review for emergency clinicians. Am J Emerg Med. 2023;69:180–7. doi: 10.1016/j.ajem.2023.04.029 37163784

[3] Proctor M, Carter N, Barker P. Community assault--the cost of rough justice. S Afr Med J. 2009;99(3):160–1. 19563091

[4] Traynor MD Jr, Laing GL, Bruce JL, Hernandez MC, Kong VY, Rivera M, et al. Mob Justice in South Africa: a comparison of blunt trauma secondary to community and non-community assaults. Injury. 2020;51(8):1791–7. doi: 10.1016/j.injury.2020.04.014 32475650

[5] Bosch X, Poch E, Grau JM. Rhabdomyolysis and acute kidney injury. N Engl J Med. 2009;361(1):62–72. doi: 10.1056/NEJMra0801327 19571284

[6] Michelsen J, Cordtz J, Liboriussen L, Behzadi MT, Ibsen M, Damholt MB, et al. Prevention of rhabdomyolysis-induced acute kidney injury - A DASAIM/DSIT clinical practice guideline. Acta Anaesthesiol Scand. 2019;63(5):576–86. doi: 10.1111/aas.13308 30644084

[7] Lewis C, Wood D. Interpersonal violence as a major contributor towards the skewed burden of trauma in KwaZulu-Natal, South Africa. S Afr Med J. 2015;105(10):827–30. doi: 10.7196/SAMJnew.8380 26428586

[8] Aborode AT, Ajagbe AO, Fasawe AS. Mob killing and its impact on public health in Africa. Med Confl Surviv. 2023;39(3):258–63. doi: 10.1080/13623699.2023.2240163 37496381

[9] Herbst CI, Tiemensma M, Wadee SA. A 10-year review of fatal community assault cases at a regional forensic pathology facility in Cape Town, South Africa. S Afr Med J. 2015;105(10):848–52. doi: 10.7196/SAMJnew.8274 26428591

[10] Skinner DL. Traumatic rhabdomyolysis (crush syndrome) in the rural setting. S Afr Med J. 2012;102(5):271–2; author reply 272. doi: 10.7196/samj.5802 22554325

[11] Chalya PL, Ngayomela IH, Rambau PF, Kahima KJ, Kapesa A, Ngallaba SE. Mob justice as an emerging medico-legal, social and public health problem in north-western Tanzania: a need for immediate attention. Tanzania J Health Res. 2015;17(1).

[12] Nivette AE. Institutional ineffectiveness, illegitimacy, and public support for vigilantism in Latin America. Criminology. 2016;54(1):142–75. doi: 10.1111/1745-9125.12099

[13] Gupta I. Mob violence and vigilantism in India. World Affairs: The Journal of International Issues. 2019;23(4):152–72.

[14] Malik S, Waseem MA, Khan U, Hussain N. The menace of mob justice: an analysis of legal provisions and high-profile cases in Pakistan.

[15] Obasanjo SB, Ifah SS, Osisiogu UC. Patterns and causes of mob justice in Nigeria. Fuoye J Criminol Security Studies. 2023;2(1).

[16] McMahon GM, Zeng X, Waikar SS. A risk prediction score for kidney failure or mortality in rhabdomyolysis. JAMA Intern Med. 2013;173(19):1821–8. doi: 10.1001/jamainternmed.2013.9774 24000014 PMC5152583

[17] Yılmaz S, Cetinkaya R, Ozel M, Tatliparmak AC, Ak R. Enhancing triage and Management in earthquake-related injuries: the SAFE-QUAKE scoring system for predicting dialysis requirements. Prehosp Disaster Med. 2023;38(6):716–24. doi: 10.1017/S1049023X23006453 37789711

[18] Lovallo E, Koyfman A, Foran M. Crush syndrome. African J Emerg Med. 2012;2(3):117–23. doi: 10.1016/j.afjem.2012.05.005

[19] Khan S, Neradi D, Unnava N, Jain M, Tripathy SK. Pathophysiology and management of crush syndrome: A narrative review. World J Orthop. 2025;16(4):104489. doi: 10.5312/wjo.v16.i4.104489 40290606 PMC12019140

[20] Jha V, Al-Ghamdi SMG, Li G, Wu M-S, Stafylas P, Retat L, et al. Global economic burden associated with chronic kidney disease: a pragmatic review of medical costs for the inside CKD Research programme. Adv Ther. 2023;40(10):4405–20. doi: 10.1007/s12325-023-02608-9 37493856 PMC10499937

[21] Hsu CC, Sandford BA. The Delphi technique: making sense of consensus. Pract Assess Res Eval. 2007;12(1):10.

[22] Kenyon CC, Palakshappa D, Feudtner C. Logic models--tools to bridge the theory-research-practice divide. JAMA Pediatr. 2015;169(9):801–2. doi: 10.1001/jamapediatrics.2015.1365 26214604

[23] Fereday J, Muir-Cochrane E. Demonstrating Rigor using thematic analysis: a hybrid approach of inductive and deductive coding and theme development. Int J Qual Methods. 2006;5(1):80–92. doi: 10.1177/160940690600500107

[24] Powell C. The Delphi technique: myths and realities. J Adv Nurs. 2003;41(4):376–82. doi: 10.1046/j.1365-2648.2003.02537.x 12581103

[25] Dalkey NC, Brown BB, Cochran S. The Delphi method: an experimental study of group opinion. Santa Monica, CA: Rand Corporation. 1969.

[26] Haines N, Doucet JJ. Severe crush injury in adults. UpToDate. 2021.

[27] Knottenbelt JD, Van der Spuy JW. Traumatic haemothorax--experience of a protocol for rapid turnover in 1,845 cases. S Afr J Surg. 1994;32(1):5–8. 11218443

[28] Sawhney JS, Kasotakis G, Goldenberg A, Abramson S, Dodgion C, Patel N, et al. Management of rhabdomyolysis: a practice management guideline from the Eastern Association for the Surgery of Trauma. Am J Surg. 2022;224(1 Pt A):196–204. doi: 10.1016/j.amjsurg.2021.11.022 34836603

[29] Rodríguez E, Soler MJ, Rap O, Barrios C, Orfila MA, Pascual J. Risk factors for acute kidney injury in severe rhabdomyolysis. PLoS One. 2013;8(12):e82992. doi: 10.1371/journal.pone.0082992 24367578 PMC3867454

[30] Liu C, Yuan Q, Mao Z, Hu P, Wu R, Liu X, et al. Development and validation of a model for the early prediction of the RRT requirement in patients with rhabdomyolysis. Am J Emerg Med. 2021;46:38–44. doi: 10.1016/j.ajem.2021.03.006 33714053

[31] Xiong Y, Shi H, Wang J, Gu Q, Song Y, Kong W, et al. Predictive model for assessing the prognosis of rhabdomyolysis patients in the intensive care unit. Front Med (Lausanne). 2025;11:1518129. doi: 10.3389/fmed.2024.1518129 39867923 PMC11759279

