# Supplementary material for: Crush syndrome diagnosis and management in resource-constrained settings: A Delphi study
Source: PLoS One. 2025 Sep 2;20(9):e0331596. doi: 10.1371/journal.pone.0331596 (PMC12404363; doi:10.1371/journal.pone.0331596)
Supplement: S1 Appendix — (DOCX) [file pone.0331596.s001.docx]

**Crush Syndrome Qualitative Interview Guide**

**INTERVIEW GUIDE**

1. **Introductions.** Please describe your clinical training, background, and the clinical environment in which you practice.
   1. What is your personal experience in caring for patients with crush injury?
2. **Definitions.** This study focuses on the diagnosis and management of crush injury. First, let us focus on definitions
   1. Where did you first learn about the conditions of crush injury and crush syndrome?
   2. Imagine you have a trainee working with you today. How would you define the condition of crush injury to them?
   3. What about crush syndrome?
   4. What about rhabdomyolysis?
   5. Do you consider crush syndrome to be a spectrum of disease, and if so, how would you further subclassify this condition?
   6. What criteria do you use to define acute kidney injury?
3. **Experience.** We would like to better understand the clinical context. Please tell me about the last patient you saw with crush syndrome and describe the nature of their injury and clinical course.
   1. Where and how often do you encounter cases of crush injury?
   2. Please describe the injury mechanisms and general demographic characteristics of the patients with crush injury you have cared for.
   3. What kinds of outcomes are you seeing for patients with crush injury?
4. **Challenges.** What are some of the challenges you have encountered in caring for patients with crush injury?
   1. If you had a chance to improve outcomes for patients with crush injury, where would you focus your attention?
5. **Diagnosis and Prognostication.** Now we will focus on specifics around diagnosis and prognostication.
   1. Tell me about your experience diagnosing patients with crush injury.
      1. What clinical and laboratory data do you use to make the diagnosis?
      2. What tools and resources do you use to support the diagnosis?
      3. In an ideal world, what additional information would you have available?
   2. What information do you use to help prognosticate for patients with crush injury regarding the development and severity of crush syndrome?
      1. What clinical and laboratory data do you use to assist with prognostication?
      2. In an ideal world, what additional information would you have available?
6. **Treatment and disposition.** What treatments do you administer when you suspect crush syndrome?
   1. How do you determine disposition for patients with crush injury and/or crush syndrome?
7. **Clinical Prediction Tools.** Have you incorporated any clinical prediction tools, protocols or algorithms to help guide clinical care for patients with crush injury? If so, please describe.
   1. Do you believe a clinical prediction tool for diagnosis and prognostication of crush syndrome would be helpful? Why or why not? Please describe what this might look like.
8. **Follow up.** Are you willing to be contacted in the future to participate in phase 2 of this study? This will involve responding to a series of 3 surveys as part of an expert consensus process.
