# Supplementary figures and images for: Crush syndrome diagnosis and management in resource-constrained settings: A Delphi study
Source: PLoS One. 2025 Sep 2;20(9):e0331596. doi: 10.1371/journal.pone.0331596 (PMC12404363; doi:10.1371/journal.pone.0331596)

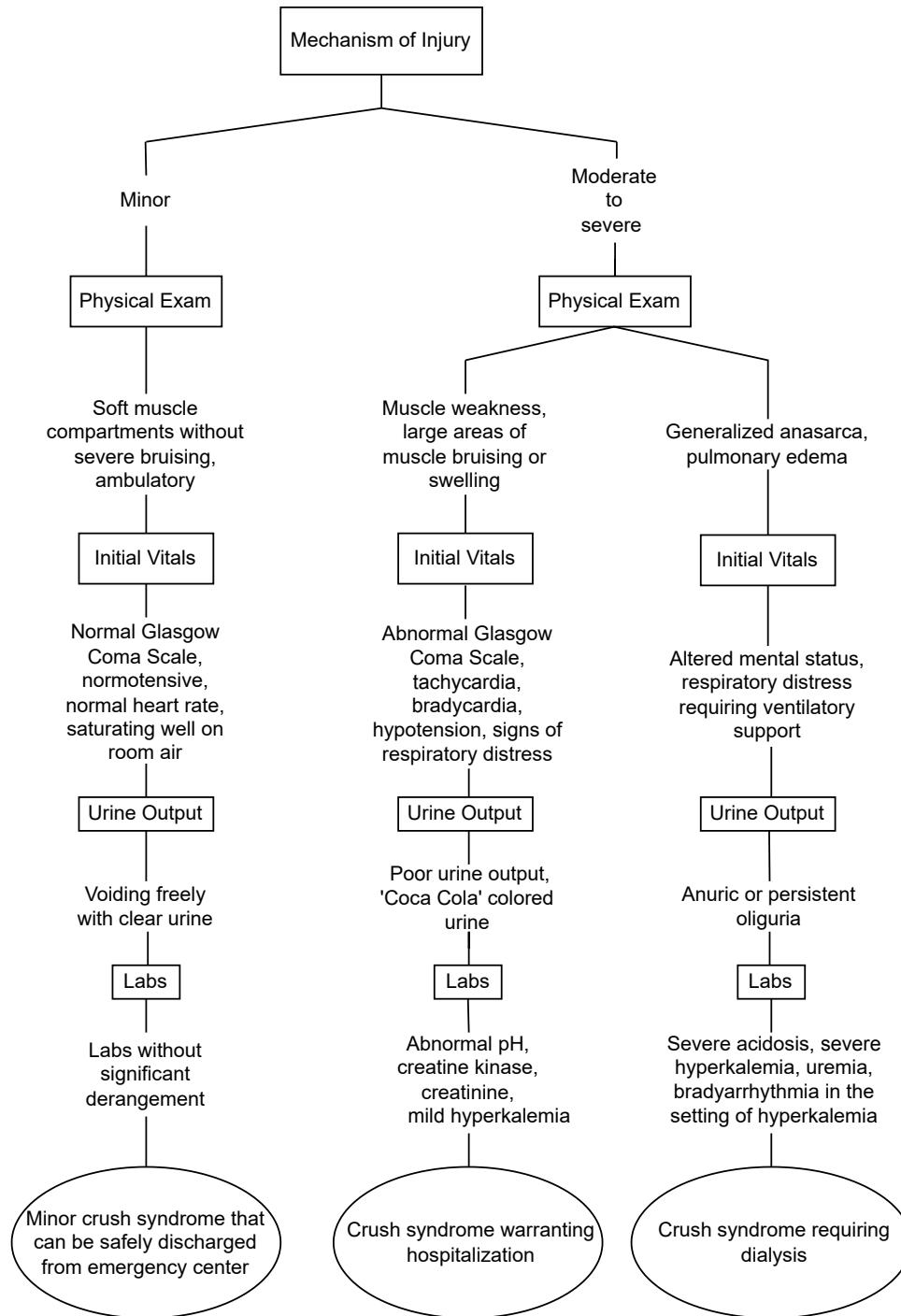

Supplement: S2 Appendix — (PDF) [file pone.0331596.s002.pdf]
